# The effectiveness of cyproheptadine as a treatment option for cats with eosinophilic airway inflammation

**DOI:** 10.18849/ve.v11i3.747

**Published:** 2026-09-17

**Authors:** Jillian Seeman, Rachael Kreisler, Jeffrey Norris

**Affiliations:** Midwestern University United States

**Keywords:** AIRWAY HYPERSENSITIVITY, BRONCHOCONSTRICTION, CYPROHEPTADINE, EOSINOPHILIC AIRWAY DISEASE, FELINE AIRWAY DISEASE, SEROTONIN

## Abstract

**PICO question:**

In cats with eosinophilic airway inflammation does cyproheptadine compared with no treatment result in a reduction in severity of clinical signs?

**Clinical bottom line:**

**Category of research:**

Treatment.

**Number and type of study designs reviewed:**

Four studies were evaluated: the first was a randomised, blinded, placebo-controlled, crossover study, the second was a randomised crossover study, the third was a non-randomised, controlled study, and the fourth a non-randomised, controlled study.

**Strength of evidence:**

Moderate.

**Outcomes reported:**

In two of the four studies it was found that cyproheptadine was not useful in resolving the symptoms of feline airway inflammation. The third study showed that the concentration of cyproheptadine that completely blocked contractile effects of serotonin did not successfully cause a significant effect on contraction caused by muscarinic stimulation. The fourth study provided evidence that cyproheptadine could reduce serotonin-induced bronchoconstriction.

**Conclusion:**

There is no current evidence to support that cyproheptadine monotherapy has any immediate benefit over no treatment for feline eosinophilic airway inflammation, and its use is not supported for this indication.

How to apply this evidence in practice

The application of evidence into practice should take into account multiple factors, not limited to: individual clinical expertise, patient’s circumstances and owners’ values, country, location or clinic where you work, the individual case in front of you, the availability of therapies and resources.

Knowledge Summaries are a resource to help reinforce or inform decision making. They do not override the responsibility or judgement of the practitioner to do what is best for the animal in their care.

## Clinical scenario

A cat presents to the clinic for the onset of bronchoconstrictive airway disease, but glucocorticoids and beta-2 agonists are contraindicated due to comorbidities or lack of access to medications.

## The evidence

The findings from four relevant studies, Schooley et al. (2007), Reinero et al. (2005), Padrid et al. (1995), and Reiche & Frey (1983), were evaluated to answer this PICO question.

Two of the studies (Reiche & Frey, 1983, Padrid et. al., 1995) investigated the effects of cyproheptadine on pulmonary resistance. Reiche & Frey (1983) determined that a dose of 16.4 μg/kg administered IV reduced serotonin-induced bronchoconstriction by 50%, the study endpoint. Cyproheptadine did not reduce pulmonary resistance induced by BGA in the study by Reinero et al. (2005). In the in vitro model used by Padrid et al. (1995), pretreatment with cyproheptadine reduced the contraction of tracheal and bronchial smooth muscles harvested from cats sensitised to *A. suum* antigen.

Two of the studies also investigated (Schooley et. al., 2007, Reinero et al., 2005) the effects of cyproheptadine on inflammatory parameters, including pulmonary infiltrates and allergen-specific immunoglobulin levels. In Schooley et al. (2007), no differences between control and cyproheptadine-treated cats were found regarding [IgA, IgE, and IgG] [eosinophils] [histamine] [serotonin]. Reneiro et al. (2005) similarly found no differences for [IgA and IgG] and [eosinophils], as well as [CD4+, CD5+, CD8+, CD21+] lymphocytes were identical between cyproheptadine-treated cats and controls. Both studies found no difference between the numbers of eosinophils in BALF collected from cyproheptadine-treated and control cats. Schooley et al. (2007) also reported no differences between histamine and serotonin levels in BALF collected from control and cyproheptadine-treated cats.

The conclusions from these studies regarding the efficacy of cyproheptadine in physiology related to clinical signs vary considerably. Reiche & Frey (1983) provided evidence that cyproheptadine reduced serotonin-induced bronchoconstriction, which was consistent with the in vitro results provided by Padrid et al. (1995), and might be expected to reduce clinical signs associated with bronchoconstriction. However, Schooley et al. (2007) and Reinero et al. (2005) provided contradictory evidence that the use of cyproheptadine in cats with airway disease would not be expected to reduce clinical signs as it was ineffective at reducing inflammation and did not reduce pulmonary resistance resulting from the bronchoconstriction induced with BGA at the doses administered.

## Summary of the evidence

**Padrid et al. (1995):** Cyproheptadine-induced attenuation of type-I immediate-hypersensitivity reactions of airway smooth muscle from immune-sensitised cats **Aim: **To study the effect of serotonergic inhibition by cyproheptadine on the responsiveness of tracheal smooth muscle (tsm) strips and epithelium-intact third-generation bronchial rings from immune-sensitized (*Ascaris suum*) cats after exposure to antigen.

Population:	Male and female mixed-breed cats between 3.5–4.5 kg.
Sample size:	10 cats each in control and sensitised groups.
Intervention details:	14 male cats and 7 female cats.Skin testing: Reaction to intradermal administration of 0.05 mL of *Ascaris. suum *(*A. suum*) antigen (1,000 protein nitrogen units (PNU) /ml) was the experimental variable. Reaction to intradermal administration of histamine (1:100,000) was the positive control. Reaction to intradermal administration of saline was the negative control. Performed prior to and 2 weeks after the sensitisation protocol. Sensitisation protocol: Administration of 0.05 ml of emulsion containing a 1:1 mixture of adjuvant and *A. suum *antigen (0.1%) in the semimembranosus muscles of each cat in the sensitised group Administration was repeated 14 days later. Measurement of lung resistance: Anaesthesia of the cats in the sensitised group was induced with sodium thiamylal (10–15 mg/kg, IV) with supplemental doses (3–5 mg/kg, IV) given at 15 to 30 minute intervals to maintain anaesthesia. Paralysis was induced by administration of vecuronium hydrobromide (0.1–0.2 mg/kg, IV). Each cat was instrumented with a 14 cm x 5 mm cuffed endotracheal tube connected and a 12G2 catheter placed into their pleural space through either the 7th or 8th intercostal space. A differential pressure transducer placed in parallel between the catheter and endotracheal tube was used to measure transpulmonary pressure. A pneumotachograph was placed in series between the endotracheal tube and a second differential pressure transducer to measure airflow, tidal volume, and lung resistance, airways suctioned to remove mucus. Airway resistance was measured prior to and for 30 minutes following exposure to *A. suum *antigen (0.01%, was nebulised for 1 minute). All cats were recovered after the procedure. Chronic antigen challenge exposure: Cats in the sensitised group were put in a nebulisation chamber and exposed to 0.01% *A. suum *antigen for a maximum of 5 minutes. Cats having signs of respiratory distress earlier than 5 min were immediately removed from the chamber. The exposure was repeated 3/wk for 6 weeks. Cats assigned to the control group were also put in a nebulisation chamber but were exposed to saline solution. The exposure was repeated 3/wk for 6 weeks. Pulmonary smooth muscle contractility: Three days after the end of the chronic antigen challenge exposure cats were euthanised under deep anaesthesia by injection of saturated potassium solution. The trachea's were immediately excised and stored in a physiologically buffered solution (K-H perfusate) with oxygen. Strips of epithelium-denuded tracheal smooth muscle (TSM) and epithelium-intact bronchial smooth muscle (BSM) were cut and connected to force transducers in perfusion chambers containing K-H perfusate. The contractile forces generated by TSM and BSM strips harvested from cats in both the sensitised and control groups were determined in response to *A. suum *antigen (0.01%) followed by 63 mM KCl. Following incubation for 20 minutes with A-64077 (100 μM, 5-lipoxygenase antagonist), the contractile forces generated by TSM and BSM strips harvested from cats in the sensitised group was determined in response to *A. suum *antigen (0.01%) followed by 63 mM KCl. Following incubation for 20 minutes with cyproheptadine (100 μM), the contractile forces generated by tracheal smooth muscle (TSM) and bronchial smooth muscle (BSM) strips harvested from cats in the sensitised group was determined in response to *A. suum* antigen (0.01%) followed by 63 mM KCl, and acetylcholine (1 mM to 1 pM) followed by 63 mM KCl.
Study design:	Non-randomised, controlled.
Outcome Studied:	In vitro study of the contractility of TSM and BSM to *A. suum *antigen alone or following pretreatment with A64077 or cyproheptadine. In vitro study of the contractility of TSM and BSM to acetylcholine alone or following pretreatment with cyproheptadine. In vitro study of the serotonin concentrations in perfusates following treatment of TSM with *A. suum *antigen alone for 10 min or following pretreatment with A64077 or cyproheptadine.
Main Findings (relevant to PICO question):	Contraction of TSM harvested from cats in the sensitised group in response to *A. suum *was reduced following pretreatment with cyproheptadine (43 ±18% KCl) compared to untreated tissue (169 ± 18% KCl, P < 0.001). Contraction of BSM harvested from cats in the sensitised group in response to *A. suum *antigen was reduced following pretreatment with cyproheptadine (16 ± 14% KCl) compared to untreated tissue (81 ± 27% KCl, P < 0.05). Compared to untreated tissue, the dose response of TSM or BSM harvested from cats in the sensitised group to acetylcholine was not significantly different following pretreatment with cyproheptadine. Following exposure to *A. suum* antigen, the amount of serotonin released into perfusates by untreated TSM, TSM pretreated with A-64077, or TSM pretreated with cyproheptadine was 12 ± 5 nM (n = 3), 9 +/– 3 (n = 3), and 18 +/– 5 (n = 2), respectively.
Limitations:	Small population of cats studied. Short time of evaluation. The degree of response by the tissues used in the experiments, which were isolated from euthanised animals, compared to that in live tissue was not determined.

**Reiche & Frey (1983):** Antagonism of the 5-HT-induced bronchoconstriction in the cat **Aim: **To compare the bronchodilator effects of ketanserin, cyproheptadine, clenbuterol, and aminophylline.

Population:	Cats of both sexes between 1.9–4.0 kg.
Sample size:	48 cats.
Intervention details:	Serotonin-induced bronchoconstriction groups (29 cats total): Ketanserin: 5–6 cats R-50970: 5–6 cats Cyproheptadine: 5–6 cats Clenbuterol: 5–6 cats Theophylline: 5–6 cats Carbachol-induced bronchoconstriction groups (19 cats total): Ketanserin: 5 cats R-50970: 5 cats Cyproheptadine: 2 cats Clenbuterol: 6 cats Theophylline: unknown number.Surgical preparation Anaesthesia was induced with chloralose (intraperitoneal, 0.26 mmol/kg). Intravenous (IV) catheter was placed in one femoral vein through which all drugs were subsequently administered. Cats were vagotomised. Cats were paralysed with suxamethonium (0.44 μmol/kg IV initially followed by 0.073–0.11 μmol/kg intravenous continuous rate of infusion (IV-CRI) for maintenance. Cats were ventilated throughout the experiment at a rate of 12 breaths/min and an inspiratory tracheal pressure of 0.98 ± 0.08 kPa. Cats were instrumented for evaluation of blood pressure from a carotid artery, central venous pressure from a jugular vein, airway pressure, and heart rate (via electrocardiogram (ECG)). Bronchoconstriction administration Serotonin was infused into a femoral vein at rates of 0.043_–_17 μmol/kg/min. Carbachol was infused into a femoral vein at rates of 2.7–7.l nmol/kg/min. Bronchodilator administration All bronchodilators were administered IV into a femoral vein. In one case, a bronchodilator (not specified) was administered subcutaneously. There was at least a thirty-minute interval between administration of each bronchodilator.
Study design:	Non-randomised, controlled.
Outcome Studied:	Dose of bronchodilator required to reduce bronchoconstriction induced by either serotonin or carbachol by 50%. Duration of action of bronchodilators. Changes in heart rate, blood pressure, and central venous pressure in response to bronchodilators.
Main Findings (relevant to PICO question):	A dose of 16.4 μg/kg (geometric mean, range of 1 SD = 9.3–29) of cyproheptadine reduced serotonin induced bronchoconstriction by 50%. In the 2 cats treated with cyproheptadine following bronchoconstriction with carbachol, the doses to reduce bronchoconstriction by 50% were 0.3 and 1.5 μg/kg. The duration of action of cyproheptadine in cats with serotonin-induced bronchoconstriction was 42 minutes (geometric mean, range of 1 SD = 32–53). Cyproheptadine did not alter blood pressure or heart rate in this study.
Limitations:	Only 2 cats were assigned to the carbachol portion of the study, making it difficult to draw conclusions about the efficacy of the drug in response to this muscarinic antagonist. The physiological bronchoconstrictors causing eosinophilic airway disease in cats are undefined and may depend on allergens, which limits the extent to which these results may be generalised. The degree of bronchoconstriction induced in this study compared to feline eosinophilic airway disease was not addressed, and therefore the clinical relevance of a 50% reduction in bronchoconstriction cannot be ascertained. Bronchodilators were administered intravenously, which would not account for reduced bioavailability due to alternate routes for routine use by clients. Blood concentrations of the drugs were not determined.

**Reinero et al. (2005):** Effects of drug treatment on inflammation and hyperreactivity of airways and on immune variables in cats with experimentally induced asthma **Aim: **To compare the effects of an orally administered corticosteroid (prednisone), an inhaled corticosteroid (flunisolide), a leukotriene-receptor antagonist (zafirlukast), on the asthmatic phenotype in cats with experimentally induced asthma.

Population:	Purpose-bred cats between the ages of 12–14 months who expressed an asthmatic phenotype following experimentally-induced asthma.
Sample size:	6 cats.
Intervention details:	3 male cats and 3 female cats.Induction of asthma and confirmation of asthmatic phenotype was done by administering Bermuda grass allgeren (BGA) at differential intervals as well as by different routes (subcutaneously and intranasal) and then observing for allergic response after repeated exposure.Treatments Each cat was randomly assigned to initially receive 1 of 5 drugs (including control). Each drug was administered for 2 weeks, then followed by a washout period of 4 weeks. Each cat received all 5 drugs during the course of the study, including: Prednisone (5 mg, PO, q 12 hr). Flunisolide (250 μg, inhaled, q 12 hr). Zafirlukast (10 mg, PO, q 12 hr, LT-receptor antagonist). Cyproheptadine (2 mg, PO, q 12 hr, antiserotonergic drug). Flour placed in No. 4 gelatin capsule (PO, q 12 hr, control).
Study design:	Randomised crossover.
Outcome Studied:	Cellular composition of BALF: Number of eosinophils expressed as percentage. Airway hyperresponsiveness: Dose of methacholine (EC200R_L_) expressed in mg/mL, with higher doses indicating a decrease in airway hyperreactivity. Serum lymphocyte phenotype: Percent of lymphocytes CD4+, CD5+, CD8+, and CD21+. Serum content of BGA-specific IgE reported as optical density. Serum and BALF content analysed for BGA-specific IgG and IgA.
Main Findings (relevant to PICO question):	The percentage of eosinophils in the cellular composition of BALF was significantly different for prednisone (5 ± 2.3%) and flunisolide (2.5 ± 1.7%), P = 0.007 among treatments for the 6 cats as compared to control (33.7 ± 11.1%), but not cyproheptadine (20.3 ± 1.7%). Airway hyperresponsiveness did not differ significantly among any treatments, with a mean (± standard error (SE)) EC200R_L_ of 4.6 ± 4.1 mg/mL for cyproheptadine as compared to 1.7 ± 1.5 mg/mL for control. Lymphocyte phenotype percentages were not different for any treatment group, with mean (± SE) for cyproheptadine (control). CD4+ 25.6 ± 2.9 mg/mL (28.5 ± 1) CD5+ 48.1 ± 10.0 mg/mL (51.5 ± 2) CD8+ 20.0 ± 3.6 mg/mL (22.1 ± 1) CD21+ 23.7 ± 3.9 mg/mL (17.0 ± 2). The level of allergen-specific IgE in the serum was significantly lower in the prednisone group (25.5 ± 4%) compared to the control group (63.6 ± 12.9%; P < 0.05). However, the level in the cyproheptadine group (45 ± 9.6%) was not significantly different from the control. Serum and BALF content of allergen-specific IgG and IgA did not show any significant differences between treatments.
Limitations:	Small sample size leading to a lack of precision and inability to examine subgroups. Induced disease model. The statistical method used is meant for normally distributed data, which is unlikely with 6 samples and no tests for normality were described. Does not state if clinicians were blinded. Airway hyperresponsiveness assessed via methacholine challenge rather than antigen challenge. Use of prednisone at approximately 1 mg/kg q 12 hrs rather than prednisolone may have been a submaximal dose, because prednisone is poorly absorbed and metabolized to the active drug (prednisolone) in cats. Use of fixed, low amounts for cyproheptadine (2 mg/cat twice daily (BID)). Subjective data regarding respiratory rate or respiratory effort may have been beneficial to the description of treatment effects. Drug concentrations in circulation were not measured. Lack of evaluation of absolute immune cell counts.

**Schooley et al. (2007):** Effects of cyproheptadine and cetirizine on eosinophilic airway inflammation in cats with experimentally induced asthma **Aim: **To determine whether oral administration of cyproheptadine or cetirizine blocks the action of serotonin and histamine, respectively, and results in diminished eosinophilic airway inflammation in cats with experimentally induced asthma.

Population:	Purpose-bred sexually intact male cats, aged 6–9 months, that weighed 4.4–5 kg. The cats were confirmed to have a negative intradermal skin test for Bermuda Grass Allergen (BGA) and had < 5% eosinophils in BALF (bronchoalveolar lavage fluid).
Sample size:	9 cats.
Intervention details:	Cats were randomly assigned to a group; each group received the treatment for one week, followed by a one-week washout period and then given the next therapeutic drug. This cycle was repeated until all 9 cats received each drug. Cyproheptadine 8 mg orally every 12 hours for one week. Cetirizine 5 mg orally every 12 hours for one week. Placebo, given in a gelatine capsule orally every 12 hours. These cats were also administered a BGA aerosol challenge 48 hours before weekly sample collection. Prior to and following each 1-week treatment period, blood and BALF samples were collected.
Study design:	Randomised, blinded, placebo-controlled crossover.
Outcome Studied:	The results of this study were objective. The presence of eosinophils in BALF was measured after the course of treatment and compared to pretreatment baseline values. The BGA-induced immunoglobulins (IgE, IgG, and IgA) concentrations in serum and BGA-specific IgG and IgA concentrations in the BALF were measured. Histamine and serotonin concentrations within the plasma and BALF using a fluorometric method were recorded.
Main Findings (relevant to PICO question):	The means ± SD for the number of eosinophils in BALF: Placebo group: 40 ± 22% Cyproheptadine group: 27 ± 16% Cetirizine group: 31 ± 20%. BGA-specific IgE, IgG, and IgA concentrations in serum and BGA-specific IgG and IgA concentrations in BALF were evaluated and showed no statistical difference between treatment groups (P > 0.05). Plasma and BALF histamine concentrations showed no statistical difference between treatment groups (P > 0.05). Plasma and BALF serotonin concentrations showed no statistical difference between treatment groups (P > 0.05). These results conclude that cyproheptadine and cetirizine were not effective in decreasing eosinophilic airway inflammation and should not be used as a monotherapy.
Limitations:	This study had a small sample size limited to only male cats. This study did not have any subjective data to describe the patients' clinical symptoms and whether or not they improved on the treatment symptomatically. The drug concentrations in circulation were not measured. An experimental study does not replicate the complexity and variability in naturally occurring eosinophilic airway disease. It is unclear if dosage or duration was sufficient to observe a therapeutic effect. In 48% of the BALF samples, histamine concentrations were below the detection limit, making it impossible to perform a statistical analysis on this data.

## Appraisal, application and reflection

Feline eosinophilic airway disease is common in cat populations. Clinical signs observed by veterinarians and owners typically include persistent respiratory issues such as sneezing, hacking, coughing, nasal discharge or mucus production, and abnormal lower airway sounds on physical examination, including crackles or wheezes. Additionally, affected cats may exhibit increased respiratory effort or an elevated respiratory rate. Advanced diagnostics can be performed on patients suspected of having lower airway disease such as bronchial alveolar lavage or lower airway wash using sterile saline. In cases of true eosinophilic airway disease, cytologic analysis of lower airway fluid may reveal moderate eosinophilic infiltration.

Feline eosinophilic airway disease is characterised by airway hyperresponsiveness and inflammation leading to increased pulmonary resistance and progressive fibrosis (Trzil, 2020). The potencies of physiological mediators of bronchoconstriction vary by species. Pulmonary smooth muscle constriction in cats is strongly stimulated by the bioactive amine serotonin, which is released from mast cells. Based on this effect, the serotonin receptor antagonist cyproheptadine merits consideration for use in the treatment of feline eosinophilic airway disease.

Two of the studies reported here (Reiche & Fry, 1983, Padrid et al., 1995) provided direct evidence that supports this treatment consideration. Reiche & Frey (1983) found that cyproheptadine reduced pulmonary resistance caused by direct administration of serotonin following intravenous administration. Padrid et al. (1995) determined that the drug reduced constriction of tracheal and bronchial smooth muscles harvested from cats sensitised to *Ascaris suum* (*A. suum*) antigen. However, serotonin increases in tissue perfusion buffers were not detected following stimulation of the tissues with the antigen. The clinical utility of these studies is limited by their experimental nature for two primary reasons. Firstly, Reiche & Frey (1983) only examined the inhibition cyproheptadine on serotonin-induced bronchoconstriction, where as Padrid et al. (1995) did not observe the clinical effects of the drug in living cats. Secondly, neither of the models evaluated clinical signs following treatment.

Reinero et al. (2005) investigated the effects of cyproheptadine on pulmonary resistance and reported no effect in Bermuda grass allergen (BGA)-sensitised cats. Circulating levels of the drug were not determined in this study. The adult cats used in this study were administered 2 mg/kg of cyproheptadine by mouth (PO) twice daily. Norris et al. (1998) reported the pharmacokinetics of cyproheptadine in cats and found that a PO dose of 8 mg led to maximal concentrations of 419 ± 96 ng/mL (n = 6) in circulation. Reiche & Frey (1983) demonstrated that an intravenous (IV) dose of 16.5 μg/kg cyproheptadine produced a 50% reduction in bronchoconstriction. Direct comparison between the results of these studies is difficult, because it would involve assuming a linear relationship between dose and plasma concentration, ignoring differences in bioavailability between the oral and intravenous administration, and does not account for the potential plateau effect of drug efficacy. Therefore, administration of a much larger oral dose than has previously been reported may be necessary to reduce pulmonary resistance in cats.

Reinero et al. (2005) and Schooley et al. (2007) investigated immunological effects in live cats using crossover study designs. Cyproheptadine did not reduce pulmonary inflammatory markers in either study, which both included pulmonary eosinophil numbers. The lack of effect on immunological markers is to be expected as serotonin is not generally considered to strongly modulate immune function. Consistent with the results from Padrid et al. (1995), Schooley et al. (2007) did not observe increases in serotonin in the bronchoalveolar lavage fluid (BALF) from BGA-sensitised cats compared to controls.

The PICO question this Knowledge Summary aimed to answer is if adult cats with eosinophilic airway inflammation cyproheptadine results in less severe clinical signs. However, the current understanding of cyproheptadine is insufficient to recommend its use for treatment of feline eosinophilic airway disease at the dosages investigated. Schooley et al. (2007) showed no statistical difference in plasma and BALF histamine concentrations between cyproheptadine and placebo groups suggesting that there was no improvement in inflammation with cyproheptadine as a monotherapy. Reinero et al. (2005) found that the prednisone significantly reduced serum allergen-specific IgE compared to the control, whereas cyproheptadine did not show statistically significant difference. Together, these studies provide evidence that cyproheptadine is ineffective and should not be used as a monotherapy at the dosages used. Padrid et al. (1995) demonstrated that pretreatment with cyproheptadine resulted in improvement in experimentally-induced inflammatory airway disease. However, the study did not address how these findings might translate to the treatment of naturally occurring disease in cats, and therefore, does not justify the use of cyproheptadine as a monotherapy for eosinophilic airway disease. Lastly, Reiche & Frey (1983) found that a dosage of 16.4 μg/kg of cyproheptadine reduced serotonin induced bronchoconstriction by 50% for 42 minutes. However, the degree of bronchoconstriction induced in this model was not directly compared to that observed in feline eosinophilic airway disease, making the clinical relevance of a 50% reduction following administration of cyproheptadine unclear, and this study cannot justify use of cyproheptadine as a monotherapy.

While the current understanding of cyproheptadine and its effects on feline eosinophilic airway disease remain unclear at its current dosing, further investigation is necessary to establish more accurate and evidence-based treatment recommendations. Norris et al. (1998) showed that cyproheptadine has 100% bioavailability following PO administration, and dose escalation studies may be warranted. Norris et al. (1998) was not appraised due to the study measuring blood levels of the medication rather than observing clinical signs of the drugs effect on a research level. Neither Reinero et al. (2005) or Schooley et al. (2007) noted any adverse effects associated with cyproheptadine treatment of cats at PO doses studied thus far.

Identification of serotonin receptor subtypes in the bronchial and tracheal smooth muscles of cats and their affinities for other serotonin receptor inhibitors may also be warranted to determine other possible drug candidates. In eosinophilic airway disease cats with diabetes mellitus, for whom treatment with glucocorticoids or beta-agonists may alter insulin requirements, or those with cardiac disease, for whom treatment with beta-agonists may be contraindicated, further investigation of serotonin antagonists for bronchoconstriction may be beneficial.

Limitations to the above studies include that many of the studies reviewed in this manuscript analyse data derived from experimentally-induced eosinophilic airway disease rather than naturally occurring cases. Induced disease may not fully replicate the complexity of the naturally occurring condition, which would exhibit different responses to the treatments under investigation. Further studies are warranted in cats with naturally occurring eosinophilic airway disease to draw definitive conclusions about the effects of cyproheptadine. Additionally, the studies did not provide an indication of clinical signs pre- and post-treatment, nor did they specify how these signs were measured. To enhance the quality of airway disease studies and improve patient care, it is recommended to implement standardised scoring systems (e.g., scales from 1–10 or 1–5) to assess symptom severity and quality of life indicators. This will allow standardised comparison to be made at different points in treatment and allow practitioners to make informed decisions of quality of life and ongoing management. Each of the studies discussed used various experimental methods which may also pose certain limitations on the findings. Schooley et al. (2007) and Reinero et al. (2005) utilised a crossover study design, which may produce an overrepresentation of treatment effects if carryover effects occur, when the washout period is insufficient and treatment effects persist into the placebo phase. Crossover designs may also underrepresent treatment effects due to their typically shorter treatment windows, which may not allow enough time for the therapeutic benefits to fully take effect.

However, Padrid et al. (1995) and Reiche & Frey (1983) utilised non-randomised controlled studies, which carry a higher risk of confounding. Without randomisation, differences in comorbidities or overall health between groups may lead to either over- or underestimation of treatment effects due to uncontrolled variability. Additionally, These studies did not address any subjective data regarding the clinical symptoms relevant to this PICO, including sneezing, wheezing, and respiratory distress.

## Methodology

**Search Strategy:** 

Databases searched and dates covered:	Pubmed via NIH (1975–2025) CAB Abstracts via CABI digital library (1995–2025)
Search strategy:	PubMed: (Cyproheptadine) AND (Cat OR Feline) AND (Asthma OR Hypersensitivity OR Bronchoconstriction) CAB Abstracts: (cyproheptadine) AND (((cat) OR (feline)) AND ((asthma) OR (bronchoconstriction) OR (hypersensitivity)))
Dates searches performed:	20 October 2025

**Exclusion / Inclusion Criteria:** 

Exclusion:	Non-primary sources/review articles. Human patients. Laboratory animals. Non-respiratory allergic reactions.
Inclusion:	Feline patients at any age with naturally or experimentally induced eosinophilic airway disease.

**Search Outcome:** 

Database	Number of results	Excluded – human patients	Excluded – laboratory animals	Excluded – non-respiratory hypersensitivity reactions	Excluded – non-primary sources	Total relevant papers
PubMed	10	3	1	2	0	4
CAB Abstracts	9	0	0	0	6	3
Total relevant papers when duplicates removed						4

## Acknowledgements

The authors would like to acknowledge Angelina R. Demartino BS, Midwestern University College of Veterinary Medicine 2027; Riley C. Hrasky BS, Midwestern University College of Veterinary Medicine 2027; Robin G. Simul BS, Midwestern University College of Veterinary Medicine 2027; Sarah Hefferan DVM, faculty at Midwestern University; and Victoria N. Moran BS, Midwestern University College of Veterinary Medicine 2027.

## ORCiD

Jillian Seeman: 0009 0001 5121 803X

Rachael Kreisler: 0000 0002 5562 5521

Jeffrey Norris: 0009 0009 6039 1529

## Disclaimer

Knowledge Summaries are a peer-reviewed article type which aims to answer a clinical question based on the best available current evidence. It does not override the responsibility of the practitioner. Informed decisions should be made by considering such factors as individual clinical expertise and judgement along with patient’s circumstances and owners’ values. Knowledge Summaries are a resource to help inform and any opinions expressed within the Knowledge Summaries are the author's own and do not necessarily reflect the view of the RCVS Knowledge. Authors are responsible for the accuracy of the content. While the Editor and Publisher believe that all content herein are in accord with current recommendations and practice at the time of publication, they accept no legal responsibility for any errors or omissions, and make no warranty, express or implied, with respect to material contained within. For further information please refer to our Terms of Use.

## References

[BIBR-1] Norris C.R., Boothe D.M., Esparza T., Gray C., Ragsdale M. (1998). Disposition of cyproheptadine in cats after intravenous or oral administration of a single dose. American Journal of Veterinary Research.

[BIBR-2] Padrid P.A., Mitchell R.W., Ndukwu I.M., Spaethe S., Shiou P., Cozzi P., Leff A. (1995). Cyproheptadine-induced attenuation of type-I immediate-hypersensitivity reactions of airway smooth muscle from immune-sensitized cats. American Journal of Veterinary Research.

[BIBR-3] Reiche R., Frey H. (1983). Antagonism of the 5-HT-induced bronchoconstriction in the cat. Archives internationales de pharmacodynamie et de thérapie.

[BIBR-4] Reinero C.R., Decile K.C., Byerly J.R., Berghaus R.D., Walby W.F., Berghaus L.J., Hyde D.M., Schelegle E.S., Gershwin L.J. (2005). Effects of drug treatment on inflammation and hyperreactivity of airways and on immune variables in cats with experimentally induced asthma. American Journal of Veterinary Research.

[BIBR-5] Schooley E.K., McGee Turner J.B., JiJi R.D., Spinka C.M., Reinero C.R. (2007). Effects of cyproheptadine and cetirizine on eosinophilic airway inflammation in cats with experimentally induced asthma. American Journal of Veterinary Research.

[BIBR-6] Trzil J. (2020). Feline Asthma: Diagnostic and Treatment Update. The Veterinary Clinics of North America: Small Animal Practice.

